# Role of novel targeted therapies in the clinic

**DOI:** 10.1038/sj.bjc.6602605

**Published:** 2005-05-31

**Authors:** R S Herbst

**Affiliations:** 1 Department of Thoracic/Head and Neck Medical Oncology, The University of Texas MD Anderson Cancer Center, 1515 Holcombe Boulevard, Unit 432, Houston, TX 77030-4009, USA

**Keywords:** targeted therapy, antitumour, monotherapy, combination therapy, patient selection

## Abstract

The number and variety of novel, molecular-targeted agents offers realistic hope for significant advances in cancer treatment. The potential of these new treatment approaches is unquestionable, but the reality is something that only thorough clinical evaluation and experience can reveal. Clinical experience of targeted therapies is at an early stage but it is likely that we will have an increasing number of treatment options available to us in the near future. This manuscript explores our current understanding of molecular-targeted therapies and considers: *What* approach should be used? (single *vs* multitarget agents); *When* should they be administered? (identifying the optimal point for intervention); *How* should they be used? (monotherapy or combination therapy regimens); and *Who* should we be giving them to? (acknowledging the need for patient selection).

In our search for new and effective approaches to the treatment of cancer, a wide range of processes essential to tumour growth and development have been identified as novel targets for anticancer therapy. The result has been the emergence of a new generation of therapeutic agents, the molecular-targeted drugs. Among these are many agents designed to inhibit tumour cell proliferation, such as proteosome inhibitors, cyclin-dependent kinase inhibitors and farnesyl transferase inhibitors. Other agents with direct antitumour activity inhibit growth factors that play a key role in tumour cell proliferation and survival, the most prominent of these is epidermal growth factor (EGF). However, a large proportion of the molecular-targeted agents currently in development primarily affect the tumour vasculature by either preventing vessel growth (antiangiogenic agents) or by destroying existing blood vessels (vascular-disrupting agents). In short, an array of novel agents is likely to become available in the clinic in the next decade and it is important that we understand the therapeutic potential, and limitations, of these novel approaches to cancer treatment.

## CURRENT USE OF MOLECULAR-TARGETED AGENTS

The first clinically available molecular-targeted agent was imatinib mesylate (Glivec®), which exemplifies a rationally designed therapy. This agent, which inhibits BCR-ABL, platelet-derived growth factor receptor (PDGFR) and c-KIT tyrosine kinases, has rapidly become established as the gold standard therapy for chronic myeloid leukaemia and gastrointestinal stromal tumours (GIST) (Druker, 2002). To date, the other clinically available molecular-targeted agents target one of two key growth factors crucial for tumour growth and development, vascular endothelial growth factor (VEGF) and EGF, as do many of the agents currently in development.

Vascular endothelial growth factor is the most potent of the angiogenic factors that function in tumour vascular development (Hicklin and Ellis, 2005). The first commercially available antiangiogenic agent, bevacizumab (Avastin™), is a monoclonal antibody (MAb) that binds to VEGF directly, thereby inhibiting receptor binding and activation. The regulatory agency approval of bevacizumab was based on a study of patients with colorectal cancer (CRC) receiving bevacizumab in combination with irinotecan/5-fluorouracil/leucovorin, which provided the first clear demonstration of the value of inhibiting VEGF signalling to improve patient survival over chemotherapy alone (Hurwitz *et al*, 2004). This agent is currently licensed for use in combination with 5-fluorouracil-based chemotherapy for first-line treatment of patients with metastatic carcinoma of the colon or rectum in the USA, and for use in combination with 5-fluorouracil/leucovorin or 5-fluorouracil/irinotecan in Europe. In contrast to bevacizumab, the majority of the anti-VEGF inhibitors currently in development target signalling through the VEGF receptor tyrosine kinase. Tyrosine kinases are primary mediators of the transmission of extracellular signals into the cell and therefore control many crucial functions including cell differentiation, growth and migration, depending on the signal received from the specific receptor (Levitzki, 2002). Unlike MAbs, the tyrosine kinase inhibitors (TKIs) will, to a lesser or greater extent, have multitarget activity.

Epidermal growth factor binds to EGFR (HER1, erB1), inducing receptor dimerisation and activation of receptor tyrosine kinase activity, thereby influencing a range of key cellular functions such as cell growth, proliferation and apoptosis (Lockhart and Berlin, 2005). The clinical value of inhibiting EGFR signalling with TKIs has been confirmed with the EGFR TKIs gefitinib (Iressa™) and erlotinib (Tarceva™), which are approved for the treatment of patients with locally advanced or metastatic non-small-cell lung cancer (NSCLC) after failure of at least one prior chemotherapy regimen. Gefitinib's approval was based on data from two large phase II dose-randomised trials (IDEAL 1 and 2) that showed significant antitumour activity (Fukuoka *et al*, 2003; Kris *et al*, 2003). The approval of erlotinib was based on a phase III study of second- and third-line treatment in patients with advanced NSCLC that demonstrated improved survival in these patients compared with best supportive care alone (Shepherd *et al*, 2004). Further support for the targeted inhibition of EGF activity has been seen with cetuximab (Erbitux™), a monoclonal anti-EGFR antibody for the treatment of EGFR-expressing, metastatic CRC. FDA approval of cetuximab was based on a series of studies that demonstrated the clinically significant activity of this agent (Cunningham *et al*, 2004).

## UNDERSTANDING THE TOXICITY POTENTIAL OF NOVEL AGENTS

Advances in cancer treatment and management mean that a number of tumour types can be considered as chronic conditions requiring long-term management. The safety profile of therapeutic agents designed for chronic administration is therefore increasingly important. The toxic effects of conventional chemotherapies are a major limitation of their use and can have a significant adverse impact on patient quality of life. The drug regimens for cytotoxic agents are based on the need for an aggressive approach, followed by a recovery period before the next treatment. In contrast, the nature of the targeted agents offers the potential for lower toxicity as their actions may have little or no effect on other body systems. Clinical evaluation of a wide range of targeted therapies has, however, shown us that these novel agents are not without toxicity issues, and the benefit : risk ratio of each agent will require careful evaluation. It is possible that the adverse events associated with targeted therapy may be less acute as the effects of therapy are less far-reaching than chemo- or radiotherapy. The potential for more subtle toxicities with molecular-targeted agents will, however, require long-term monitoring.

One of the first antiangiogenic agents to be evaluated in patients was SU5416, an inhibitor of the VEGFR and c-KIT receptor tyrosine kinases (Fong *et al*, 1999). While this early agent advanced our understanding of the potential of this new class of drugs, its clinical application was severely limited by poor physical and pharmacokinetic properties (necessitating frequent parenteral administration in a Cremophor vehicle) and a range of adverse effects that included many cardiovascular events (Kuenen *et al*, 2002; Heymach *et al*, 2004a). The lack of demonstrable clinical efficacy seen with this agent, as well as negative data from other studies of novel agents, was therefore disappointing. While bevacizumab had demonstrated activity in patients with renal, breast and NSCLC, (Hillan *et al*, 2003; Yang *et al*, 2003; Johnson *et al*, 2004) a turning point came with the results of the phase III study of the bevacizumab/IFL combination in patients with CRC, providing the first conclusive evidence of a survival benefit with molecular-targeted antiangiogenic therapy over a conventional chemotherapy regimen alone (Hurwitz *et al*, 2004). However, since the launch of bevacizumab, healthcare providers have been warned of the increased risk of arterial thromboembolic events associated with its use. These events include transient ischaemic attacks, myocardial infarctions and angina, some of which were fatal. In patients with NSCLC, a study of bevacizumab in combination with carboplatin and paclitaxel has identified squamous cell histology as predisposing for an increased risk of bleeding and life-threatening pulmonary haemorrhage (Johnson *et al*, 2004).

The range of VEGFR TKIs currently in development seem to have varying toxicity profiles and further evaluation will elucidate those with the most promising benefit : risk ratio. It is the role of the phase I study to identify toxicity problems and despite the inevitable failures at this stage many anti-VEGF agents have demonstrated acceptable tolerability and have moved to a more advanced stage of clinical development. PTK787, BAY 43-9006, SU11248 and ZD6474 have generally demonstrated manageable safety profiles and the dose-limiting toxicities tend to be manageable events such as gastrointestinal toxicity, fatigue and rash. Hypertension has also been reported with some of the VEGFR TKIs and is likely to occur as a result of VEGFR inhibition. It is therefore interesting that some of the observed drug-related adverse events may act as surrogate markers of target inhibition. An example of this in another class of molecular-targeted agents is seen with EGFR inhibition, which has been associated with the development of rash, a common and dose-related observation in studies of erlotinib and cetuximab (Perez-Soler, 2003). In fact, there may be a correlation between patients who develop severe drug-induced rash and improved survival (Perez-Soler, 2003).

## CLINICAL APPLICATION OF NOVEL THERAPIES

The number and variety of novel targeted agents offers realistic hope for significant advances in cancer treatment. The ongoing clinical evaluation of these agents will reveal which offer the greatest therapeutic value, but the range of target profiles and properties poses a number of questions. Clinical experience of molecular-targeted therapies is at an early stage, but it is likely that we will have an increasing number of treatment options available to us in the near future. The potential of these new treatment approaches is unquestionable, but the reality is something that only thorough clinical evaluation and experience will answer. In the meantime, we can consider the ‘What, When, How and Who?’ of targeted therapies.

### What? − single *vs* multiple targets

The molecular-targeted agents currently in development can be described in terms of their target profile as being single- or multiple-target agents (Table 1). However, the only agents that are purely targeted against one receptor are the MAbs, as small molecule ATP-competitive agents frequently have additional off-target activities against other receptor tyrosine kinases, especially at higher doses. To date, our clinical experience is based on agents with specific primary targets, that is, the anti-VEGF MAbs bevacizumab and cetuximab, and the EGFR TKIs erlotinib and gefitinib. A number of other highly selective agents are in development, such as IMC-1C11, an anti-VEGFR-2 MAb and TKIs with VEGFR-specific activity (e.g. CEP-7055 and GW-786034), all of which have demonstrated promising efficacy and safety in early clinical studies (Posey *et al*, 2002; Pili *et al*, 2003). It could be argued that the target specificity of these agents provides a ‘clean’ approach to therapy and enables the efficacy and tolerability to be determined in the absence of effects caused by inhibition of other pathways. For the antiangiogenic agents in particular, however, this single profile may predict the need for use in combination with other agents in order to maximise the treatment effect. Indeed, the only licensed antiangiogenic agent, bevacizumab, has demonstrated a survival benefit only in combination regimens (Hurwitz *et al*, 2004).

Many of the newer agents inhibit more than one receptor tyrosine kinase and these compounds may have unique inhibition profiles. For example, ZD6474 inhibits both VEGFR and EGFR tyrosine kinase activity, and therefore has the ability to block two key processes in tumour development (Wedge *et al*, 2002). Other agents possess different receptor tyrosine kinase inhibition profiles, such as PTK787, an oral inhibitor of VEGFR tyrosine kinase that also inhibits the related kinases PDGF-*β* receptor and c-Kit (Wood *et al*, 2000; Drevs *et al*, 2003; George *et al*, 2003; Reardon *et al*, 2003). BAY 43-9006 is a potent inhibitor of Raf-1 (the Ras/Raf signalling pathway being an important mediator of tumour cell proliferation and angiogenesis), VEGFR and PDGFR TKIs. These agents can therefore inhibit both tumour cell proliferation and angiogenesis. Clinical evaluation of ZD6474, PTK787 and BAY 43-9006 has been promising, with evidence of tumour regression observed in a variety of tumour types, including NSCLC, renal cell carcinoma, CRC, melanoma, thyroid, sarcoma and pancreas (Minami *et al*, 2003; Ahmad and Eisen, 2004; Ratain *et al*, 2004; Siu *et al*, 2004; Veronese *et al*, 2004; Wilhelm *et al*, 2004). SU11248 inhibits a still wider range of receptor tyrosine kinases, with *in vitro* activity against VEGFR-1, VEGFR-2, Flt-3, PDGFR, c-Kit and CSF-1 receptor tyrosine kinase activity (Abrams *et al*, 2003a, 2003b). The antitumour activity of this agent has been demonstrated in a study of patients with GIST. While the efficacy and safety profile of SU11248 has been deemed sufficient to warrant further investigation, the incidence of adverse events has been greater than with imatinib, highlighting the potential for multitargeted approaches to be associated with a comparatively high toxicity profile.

Agents that can target several pathways in tumour growth are highly attractive, potentially offering the benefits of combined therapy within a single agent. Such combined effects may, however, make it difficult to demonstrate the relative benefits of targeting each pathway and the possibility of additive toxicity will require thorough evaluation.

### When? – identifying the optimal point of intervention

As with all anticancer strategies, detecting and treating tumours at the earliest possible stage is likely to result in the best outcome for the patient. This may be of particular importance for the antiangiogenic agents, where prevention of tumour development, rather than frank regression, is the anticipated effect. Early intervention with an antiangiogenic agent could, in theory, prevent metastatic spread and while clinical demonstration of this effect is likely to prove difficult, preclinical evidence is compelling (Drevs *et al*, 2002; Wu *et al*, 2004). An additional consideration is the knowledge that VEGF is a key driver of angiogenesis early in tumour development, but a range of other proangiogenic factors, such as bFGF and PDGF, are likely to contribute to vascular development at later stages (Relf *et al*, 1997; Pavlakovic *et al*, 2001; Kerbel, 2004). Patients with later stage disease may benefit from intervention with vascular-disrupting agents, which function by exploiting key differences between tumour and normal blood vessels to selectively destroy the existing tumour vasculature. Preclinical evaluation of the vascular-disrupting agent ZD6126 has shown efficacy in a range of tumour sizes, but particularly in larger tumours (Siemann *et al*, 2002).

The use of antiangiogenic agents in the adjuvant setting is another area of great potential. Surgical removal of tumours can result in the development of previously dormant metastases due to the antiangiogenic effects exerted by the dominant tumour (Holmgren *et al*, 1995). Further evaluation of the benefits of administering antiangiogenic therapy prior to surgery, or even chemotherapy, would therefore be warranted.

### How? − monotherapy or combination regimens

It is likely that the nature of the target inhibition will largely dictate whether agents will function effectively as monotherapy or whether combined therapeutic regimens will be required. Of the currently available agents, the EGFR TKIs have proved effective as monotherapy, whereas bevacizumab has demonstrated a survival benefit only in combination regimens (Hurwitz *et al*, 2004). This is likely to be due to the mechanism of action of the EGFR inhibitors, which target the tumour cells directly, whereas VEGF inhibition can be anticipated to elicit a cytostatic effect and therefore combination with conventional approaches may be required in order to eradicate the tumour. Currently available agents and developmental therapies alike are therefore being investigated in a wide range of combination regimens, sometimes to the exclusion of monotherapy studies (Table 2). PTK787 and early studies of ZD6474 have, however, shown evidence of efficacy as monotherapy (Minami *et al*, 2003; Morgan *et al*, 2003). In addition, a phase III study of SU11248 monotherapy in patients with imatinib-resistant/imatinib-intolerant GIST has recently been halted ahead of schedule as interim analysis clearly demonstrated the efficacy benefits of this approach. While it is evident that targeted agents can prove effective as monotherapy, the question is whether this efficacy can be improved upon with combination regimens.

To date, the most successful use of the antiangiogenic agents has been in combination with certain conventional chemotherapies (Hurwitz *et al*, 2004; Steward *et al*, 2004). It is possible that combined use may enable the constituent agents to be used at lower doses, but the potential drug–drug interactions and altered pharmacokinetic and biological effects of drug combinations require careful evaluation. An additional benefit of combination therapy is the observation that antiangiogenic therapy may result in a degree of normalization of blood vessels. (Jain, 2001; Willett *et al*, 2004). As tumour blood vessels are structurally and functionally abnormal, blood flow throughout the tumour is variable and areas of hypoxia are common. Vascular endothelial growth factor inhibitors have been shown to decrease interstitial pressure and increase oxygen tension, and this may assist the delivery of cytotoxic agents (Jain, 2001). In addition to complementing chemotherapy regimens, it is possible that targeted agents could enhance the therapeutic efficacy of radiation therapy as VEGF levels and tumour cell EGFR expression have been shown to increase following exposure to ionising radiation (Akimoto *et al*, 1999; Dent *et al*, 1999; Gorski *et al*, 1999). There has been little clinical evaluation of this application to date, but a wealth of preclinical studies have provided convincing evidence for this approach (Gorski *et al*, 1998; Mauceri *et al*, 1998; Gustafson *et al*, 2004; Siemann and Shi, 2004; Williams *et al*, 2004).

The specific benefits of the combined use of targeted agents is being investigated in a number of studies evaluating the combined use of these agents. A dual approach to targeting tumour cell proliferation directly by inhibiting EGF activity, plus tumour vascularization by inhibiting VEGF activity is attractive, and studies of bevacizumab plus the EGFR TKI erlotinib have shown positive results in a second/third-line study of patients with NSCLC of non-squamous cell histology (Herbst *et al*, 2005). In this study, partial responses were seen in 20% of patients, and stable disease in 65% (Herbst *et al*, 2005). Ongoing clinical evaluation of ZD6474 will elucidate the benefit of a single-agent approach to VEGFR and EGFR inhibition.

The results of the ongoing studies of single-agent therapy with multitargeted drugs and the various combination regimens currently under investigation will hopefully clarify the differential benefits of using multitargeted drugs *vs* combined use of more selective agents. In particular, the tolerability issues associated with these approaches will require careful evaluation in order to demonstrate whether it may be safer to use a combination of highly targeted agents, or one multitargeted drug.

### Who? − acknowledging the need for patient selection

Heterogeneity is manifest at a number of levels in human cancer; genetically, at the cellular level, zonally (within a tumour deposit), between tumour deposits, and between patients. An awareness and understanding of this heterogeneity is key to the development of tailored biological therapies, and could be greatly assisted by the development of better, more predictive animal models. The goal of conventional chemotherapies is to kill all rapidly proliferating cells, which accounts not only for its widespread application to all tumour types but also for its significant associated toxicity. Targeted therapies, by definition, act in a far more specific manner, inhibiting biological pathways and processes that are selectively dysregulated in tumours, thereby avoiding many of the tolerability disadvantages of conventional chemotherapy. As a result, however, it is likely that a ‘one size fits all’ approach cannot be adopted with the novel agents, and that a degree of patient selection may be required to identify the patients who are likely to benefit most from treatment.

The successes and failures of clinical studies to date highlight the need to identify specific patient types for treatment with the various targeted therapeutic approaches, and a great deal of additional investigation is required before we can claim to understand and optimise treatment. For example, despite the excellent data reported with bevacizumab plus chemotherapy in the first-line CRC study, investigation of this agent as a third-line therapy in combination with capecitabine in patients with metastatic breast cancer has shown evidence of activity (as seen by a significant increase in response rates), but no significant improvement in survival (Miller *et al*, 2005). Such evidence of biological activity that fails to translate into an overall survival benefit could be considered further evidence of the need for patient characterisation; it is likely that while specific approaches may be generally more effective in certain tumour types, subgroups of patients that demonstrate a survival benefit could be identified in a range of tumour types.

Although the activity of antiangiogenic agents should theoretically apply to all solid tumours, there appear to be key differences between patient populations. One hypothesis is that although early-stage tumours may rely on VEGF as the principal proangiogenic factor, angiogenesis in late-stage disease may be governed by a range of proangiogenic factors and there may be some redundancy of VEGF (Relf *et al*, 1997; Pavlakovic *et al*, 2001; Kerbel, 2004). It is therefore possible that VEGF is a less significant factor in late-stage, treatment-refractory breast cancer than early-stage breast cancer or other solid tumours. It is also possible that tumour types that express high levels of VEGF and its associated receptors may be particularly susceptible to antiangiogenic therapy. Microvessel density appears to be an important prognostic marker but the correlation with predicting response to antiangiogenic therapy is not so clear. For example, renal tumours are highly vascularised and would be expected to be particularly sensitive to antiangiogeneic agents, whereas other, less vascularised, tumour types may be less susceptible. It cannot, however, be presumed that a poorly vascularised tumour will be less susceptible to antiangiogenic therapy as it is well established that neovascularisation must take place for tumours to grow beyond a certain size. Indeed, there is some evidence to suggest that lower vascularity increases the response to antiangiogenic therapy (Beecken *et al*, 2001).

In patients with NSCLC, a response to EGFR TKI therapy is difficult to predict, but it is known that response does not appear to correlate with the degree of EGFR expression (Bailey *et al*, 2005). Mutations in the tyrosine kinase domain of the EGFR have been correlated with clinical responsiveness to gefitinib (Lynch *et al*, 2004; Han *et al*, 2005). Such mutations were seen to be more frequent in adenocarcinomas, in women and in Japanese patients (Lynch *et al*, 2004; Paez *et al*, 2004). Our knowledge of specific mutations that may predispose response to other agents is, however, formative and further evaluation is required. Recent phase III evaluation of gefitinib (IRESSA Survival Evaluation in Lung cancer, ISEL) showed that the increase in overall survival of treated patients was not statistically significantly greater than the placebo arm. However, in patients of Oriental origin and in patients who have never smoked, the data suggested a statistically significant difference.

Additional evidence of the need for patient screening has come from studies of bevacizumab in patients with NSCLC. While the tolerability profile of this agent is generally good, multivariate analysis has identified patients with squamous cell histology as being at higher risk of bleeding and pulmonary haemorrhage (Johnson *et al*, 2004). This adverse event has not been notable in studies of bevacizumab in other tumour types.

## CONCLUSIONS

As the clinical experience with novel anticancer agents expands, we will be faced with increasingly complex treatment decisions to accompany the increase in available treatment options. It will be important to establish how these treatments can add to, or replace, conventional cytotoxic therapy and improve on patient outcomes from both an efficacy and safety or quality of life aspect. It will also be important to understand which patients benefit most from the numerous options, so that the treatment strategy can be tailored to the individual patient and meet their needs.

The ultimate goal of cancer research has always been, and remains, to find a cure. A more realistic interim goal for patients with metastatic disease may, however, be to adopt a ‘regulatory model’ of cancer, which aims to control tumour burden and limit metastasis, thereby achieving a ‘functional cure’, rather than eradicating all tumour cells (Schipper *et al*, 1995). In this way, patients could live with cancer as a chronic disease that can be effectively managed with similar efficacy to diabetes and heart disease. The molecular-targeted agents could prove instrumental in achieving this goal by providing different methods of slowing or stopping tumour growth in a chronic treatment setting. Continued research and clinical evaluation will serve to expand our understanding and improve our ability to provide optimal, tailored therapeutic strategies with this new generation of anticancer agents.

## Figures and Tables

**Table 1:** Activity profile of molecular-targeted agents

**Single target**	**Multiple target**
Bevacizumab (anti-VEGF MAb)	BAY 43-9006 (Raf-1, VEGFR-2 and -3, and PDGFR-*β*)

Cetuximab (EGFR MAb)	PTK787 (VEGFR-1, -2 and -3, PDGFR-*β* and c-Kit)

IMC-1121b (VEGFR-2 MAb)	SU11248 (VEGFR-1, -2 and -3, Flt-3, PDGFR, c-Kit and CSF-1)
	ZD6474 (VEGFR and EGFR)
	AEE-788 (VEGFR, EGFR, erb)
	AMG 706 (VEGFR, PDGFR, c-Kit and Ret)

**Table 2:** Clinical experience of combination regimens

**Phase of study**	**Targeted therapy**	**Chemotherapy**	**Tumour type/size of study**	**Status**
III	Bevacizumab	Irinotecan/5-fluorouracil/leucovorin	Untreated metastatic CRC (*n*=813)	Complete. The addition of bevacizumab to fluorouracil-based combination chemotherapy results in statistically significant and clinically meaningful improvement in survival (Hurwitz *et al*, 2004)

II	Bevacizumab	Carboplatin/paclitaxel	Advanced/recurrent NSCLC (*n*=99)	Complete. Inclusion of bevacizumab resulted in a higher response rate compared with the control therapy (31.5 *vs* 18.8%), a longer median time to progression (7.4 *vs* 4.2 months) and a trend towards increased survival (17.7 *vs* 14.9 months) (Johnson *et al*, 2004)

I/II	PTK787	FOLFOX-4	Metastatic CRC (*n*=35)	Complete. Combined treatment was well tolerated at doses ⩽1250 mg day^−1^; adverse events at 1250 mg day^−1^ included grade 3 ataxia, thrombocytopenia and dizziness, and grade 4 neutropenia. Grade 3 expressive dysphasia and intermittent dizziness were dose limiting at 1500 mg day^−1^. Best response data for 28 evaluable patients to date show one CR, 14 PR, and nine stable disease . Estimated median overall survival for 35 patients is 16.6 months (Steward *et al*, 2004)

III	PTK787	FOLFOX-4	Metastatic CRC CONFIRM-1 (previously untreated patients): *n*=>1000 CONFIRM-2 (patients who have progressed after irinotecan-based first-line chemotherapy): *n*=>800	CONFIRM-1: analysis of progression-free survival (primary end point) showed no statistically significant improvement over FOLFOX-4 alone (unpublished data). Assessment of overall survival (secondary endpoint) ongoing CONFIRM-2: ongoing

III	Bevacizumab	Carboplatin/paclitaxel	Advanced/recurrent NSCLC (first-line) *n*=878	Complete. Analysis has demonstrated a survival benefit with bevacizumab/carboplatin/paclitaxel of 12.5 *vs* 10.2 months with chemotherapy alone (unpublished data)

II	ZD6474	Docetaxel	Locally advanced or metastatic NSCLC after failure of first- line platinum-based chemotherapy *n*=>200	Ongoing. Data from safety run-in phase have shown good tolerability with this combination (Heymach *et al*, 2004b)

II	ZD6474	Carboplatin/paclitaxel	First-line NSCLC (*n*=>200)	Ongoing. Preliminary data from the safety run-in phase show that the combination is generally well tolerated (Johnson *et al*, 2005)

II	Bevacizumab+erlotinib	Docetaxel (alone or+bevacizumab)	Refractory NSCLC (*n*=>180)	Ongoing

I/II	PTK787	Temozolomide or lomustine	Glioblastoma multiforme (*n*=60)	Ongoing. Interim data show good tolerability and promising efficacy (Reardon *et al*, 2004)

I/II	Bevacizumab+erlotinib	n/a	Stage IIIB/IV or recurrent NSCLC (*n*=40)	Complete. Eight partial responses (20%, CI 7.6−32.4%) and 26 patients with SD (65%, CI 50.2−79.8%). The median survival of 34 patients treated in the phase II part of the study was 12.6 months, with 52% of patients alive at 1 year (Sandler *et al*, 2004)

I	Bevacizumab+BAY 43-9006	n/a	Various (*n*=38)	Ongoing

CR=complete response; SD=stable disease; PR=partial response; NSCLC=non-small-cell lung cancer; CRC=colorectal cancer; n/a=not applicable.
